# The Impact of Antenatal and Postnatal Factors on the Development of the Pulmonary Microvasculature in Preterm Infants

**DOI:** 10.3390/children12050650

**Published:** 2025-05-18

**Authors:** Raluca Chirculescu, Ruxandra Viorica Stănculescu, Paul Cristian Bălănescu, Gheorghe Peltecu

**Affiliations:** 1 Department of Obstetrics and Gynecology, Carol Davila University of Medicine and Pharmacy, 020021 Bucharest, Romania; 2 Department of Pathology, Filantropia Clinical Hospital, 011132 Bucharest, Romania; 3 Department of Internal Medicine, Colentina Clinical Hospital, 020125 Bucharest, Romania; 4 Department of Obstetrics and Gynecology, Ponderas Academic Hospital, 014142 Bucharest, Romania

**Keywords:** preterm birth, microvascular pulmonary remodeling, bronchopulmonary dysplasia, chronic lung disease, alveolar simplification, oxygen therapy

## Abstract

This research aimed to assess the influence of prenatal and postnatal factors on the remodeling of the pulmonary microvasculature. **Methods**: The investigation analyzed 67 cases of preterm infants, whose lifespans ranged from 1 day to 149 days. After selecting the cases from the autopsy database, two lung tissue microarrays were created. Histological slides were stained using the hematoxylin and eosin technique to precisely capture the microscopic details. For the assessment of pulmonary microvascularization and the media layer of the vascular walls, an immunohistochemical analysis was performed utilizing CD34 and SMA markers. **Results**: Following the assessment of the quantity of capillaries positive for CD34, a negative correlation was identified between the average capillary count per alveolus and the duration of oxygen therapy. Preterm infants who developed pulmonary fibrosis exhibited an average reduction of 5.43 capillaries in comparison to other newborns. Preterm neonates born to mothers with preeclampsia exhibited an average reduction of 2.82 capillaries compared to those born to mothers unaffected by this pregnancy complication. A positive correlation was evident between increased thickness of the arteriolar media, lifespan, and the duration of oxygen therapy, as well as in those preterm infants who developed pulmonary fibrosis. **Conclusions**: Antenatal risk factors did not exert a significant impact on pulmonary vascular remodeling, whereas postnatal influences, particularly oxygen therapy, demonstrated a detrimental effect on the density of capillary structures within the alveolocapillary membrane. Premature neonates with increased thickness of the arteriolar media had a greater susceptibility to pulmonary hypertension.

## 1. Introduction

Lung development is a complex process of phases and stages that interact to achieve a fully functional respiratory system following birth. Although a term newborn is capable of successfully adapting to the extrauterine environment, the lung is an elaborate organ that continues to mature after birth by increasing the number of alveoli and reorganizing the pulmonary vascular network [1,2]. During fetal life, the lungs do not perform their essential function as organs of gas exchange. Instead, this vital role is carried out by the placenta [3]. Consequently, the fetus exhibits a remarkable ability to redirect blood circulation, bypassing the pulmonary system through the foramen ovale, the patent ductus arteriosus, and the ductus venosus. This leads to a distinctive feature of reduced blood flow and elevated vascular resistance in the fetal pulmonary vascular network [4,5]. Upon the clamping of the umbilical cord at birth, the systemic arterial pressure begins to increase, while the previously heightened pulmonary arterial pressure concurrently initiates its descent, accompanied by an increase in pulmonary blood flow [5]. This phenomenon is the result of air entering the lungs, which induces pulmonary vasodilation along with a decrease in pulmonary vascular resistance [4]. This transition is affected in the preterm neonate due to physiological and metabolic immaturity, leading to an increased necessity for neonatal resuscitation procedures [6]. Over time, as advancements in neonatal therapies have progressed, the life expectancy of premature infants born at extreme gestational ages (24–28 weeks of gestation) has shown remarkable improvement. Despite the increasing survival rate, a subset of these premature births may develop chronic lung disease, a condition influenced by incomplete pulmonary development and exacerbated by the necessity for intensive interventions to sustain respiratory function [7,8]. The most prevalent chronic respiratory condition observed in premature infants, which is associated with pulmonary microvascular abnormalities, is undoubtedly bronchopulmonary dysplasia [9]. This pulmonary condition most frequently manifests in premature neonates who are delivered during the canalicular phase of pulmonary development and is primarily characterized by alveolar simplification. Furthermore, the septal capillaries composing the alveolar–capillary membrane will also be impacted, a phenomenon known as pulmonary vascular remodeling [10,11,12]. Although alveolar simplification is a widely recognized and much more common morphological feature compared to pulmonary fibrosis, which we encountered in premature newborns who developed bronchopulmonary dysplasia before the introduction of surfactant therapy and current oxygen therapy techniques, pulmonary vascular remodeling abnormalities are a significant component of the chronic lung diseases of these preterm newborns [1]. Alongside the formation of an adequate number of alveoli (around 500 million alveoli in a fully developed lung), the existence of a refined capillary network is essential for ensuring effective gas exchange [1,13]. These two processes begin and develop simultaneously, starting during intrauterine life and continuing for several years after birth [14]. The development of intrapulmonary vascular structures occurs in a series of stages, beginning in the fifth week of gestation, which coincides with the onset of the pseudoglandular phase of pulmonary development. Although the vascular system reflects the branching pattern of the bronchial tree, the pulmonary vascular network will produce 20 percent more generations than the bronchial structure. Through the process of angiogenesis in the canalicular stage of pulmonary development, the capillary vascular network begins to take shape. Following 27 weeks of gestation, the saccular phase initiates, wherein the septa of the future alveoli begin to diminish in thickness, and the developing bilayered capillary network commences its approach toward the septal surface. The alveolar phase initiates following 36 weeks of gestation and continues beyond birth until the onset of young adulthood. At this stage of development, the capillary network undergoes maturation as the dual capillary networks converge to form a singular, monolayer capillary network that is in intimate association with the alveolar epithelial cells. As new alveolar septa arise postnatally, the simultaneous development and maturation of the septal capillary network will progress concurrently [15]. Since intrauterine lung development is a time-dependent, stadial phenomenon, it is understandable that any disruption in the process of pulmonary development will not only impact the total quantity of alveolar structures but also the density of capillaries. Due to the limited amount of research conducted on human subjects, various animal study models have been established over time to replicate the effects of impaired lung development caused by preterm birth. By far, rodents have been the most frequently utilized animal models due to their short gestational period, cost-effectiveness, and the unique pulmonary structural characteristics of being born in the saccular phase of lung development [8]. These studies have demonstrated that postnatal interventions involving oxygen and barotrauma lead to the cessation of alveolarization and pulmonary vascular development [16,17]. Furthermore, research conducted on large mammals such as baboons has led to the development of intricate models that closely replicate the clinical, imaging, and histopathological characteristics observed in premature human newborns with bronchopulmonary dysplasia. These animal subjects were born prematurely and exposed to therapeutic interventions replicating those provided to premature human newborns, resulting in the observed reduction in alveolar count and alterations in pulmonary microvascularization [18]. Furthermore, it has been observed that prolonged mechanical ventilation in preterm infants results in the disruption of the transformation of the capillary septal network from a dual capillary configuration to a singular one. This morphological characteristic of a dual capillary network is particularly distinctive during the canalicular and saccular phases of pulmonary development [1,19]. Although bronchopulmonary dysplasia has been extensively studied over the years, there are still numerous uncertainties surrounding this condition. The primary antenatal and postnatal risk factors that can influence the development of bronchopulmonary dysplasia [11] are outlined in Table 1.

The main purpose of this investigation is to explore various prenatal and postnatal risk factors associated with the development of bronchopulmonary dysplasia that may impact pulmonary microvascular growth in premature infants.

## 2. Materials and Methods

The research encompasses 67 cases retrospectively selected from the autopsy database conducted at the pathology departments of two maternity hospitals in Bucharest, Romania. Of the entire study cohort, 39 cases were selected from the National Institute for Mother and Child Health “Alessandrescu-Rusescu” between January 2018 and December 2020, while the remaining 28 cases were gathered from the Filantropia Clinical Hospital from January 2017 to December 2024. This research was conducted subsequent to obtaining favorable approvals from the ethics committees of both institutions, under registration numbers 21875/02.12.2020 and 12407/19.12.2024.

### 2.1. Study Population

#### 2.1.1. Inclusion and Exclusion Criteria

Given that term newborns experience ongoing development of pulmonary microvascularization postnatally, and considering that one of the postnatal parameters monitored in this study was incomplete pulmonary maturation, we aimed to ascertain whether the progression of pulmonary microvascularization continues in this particularly susceptible cohort of preterm neonates. Consequently, we established inclusion criteria of a gestational age below 37 weeks, indicating pulmonary immaturity, in conjunction with a lifespan of at least 24 h, thereby permitting the external environment to exert influence on the morphology of lung tissue.

All neonates who did not fulfill these criteria, in addition to neonates afflicted with pulmonary hypoplasia or diaphragmatic hernia leading to pulmonary hypoplasia, were omitted from this study.

Because the subject selection period overlapped with the COVID-19 pandemic, it is important to note that none of the cases included in the study exhibited any COVID-19 infections, nor did any of the preterm newborns born to mothers who had contracted the virus.

#### 2.1.2. Sample Size and Data Collection

The medical data were obtained from the digital archives, maternal and neonatal observation forms, and autopsy reports from both healthcare institutions over a cumulative period of 8 years (2017–2024). Subsequent to a rigorous examination of the medical data, 67 cases were selected. Relevant information regarding the mother and child was extracted from the cited medical documentation and meticulously recorded in an Excel database.

Among all the parameters documented in the database, the Apgar score requires additional clarification. This evaluation is an immediate assessment of the newborn at birth that involves quantifying 5 parameters (breathing, heart rate, skin color, muscle tone, and reflexes); each parameter is assigned a score of 0, 1, or 2 points. The evaluation is performed at 1 and 5 min following birth, and if the score is below 7, the assessment should be recorded after another 5 min [20]. Since medical data regarding the Apgar score at the 10 min was not accessible to all participants in the study, an assessment of this determination could not be carried out.

Another significant parameter conceptualized as a prenatal risk factor, which could not be documented in the database due to insufficient data from medical records, was maternal smoking and, by extension, the intrauterine exposure of the fetus to cigarette smoke.

### 2.2. Work Procedures

#### 2.2.1. Selection of Paraffin-Embedded Lung Tissue Blocks

The paraffin-embedded tissue blocks designated for this investigation consist of lung tissue fragments obtained after histopathological processing. These tissue specimens were procured during post-mortem gross examinations. Given that paraffin-embedded lung tissue blocks serve as the source for subsequent histopathological and immunohistochemical analyses, a reevaluation of their corresponding histopathological slides has been undertaken to determine the most appropriate paraffin-embedded tissue specimens.

The initial step involved retrieving from the archives (histotechs) all pulmonary tissue slides stained with hematoxylin and eosin that pertained to preterm infants encompassed within the study. This was succeeded by a thorough re-evaluation of all histological lung tissue slides using the optical microscope available at each medical institution. Histological slides older than 5 years were recut and re-stained using the conventional hematoxylin and eosin technique to precisely capture the microscopic details. From each slide, representative lung regions were selected and encircled with a marking pen under the optical microscope for subsequent biopsies.

#### 2.2.2. The Construction of Tissue Microarrays

Tissue microarrays are paraffin-embedded tissue blocks that encompass multiple histopathologically processed fragments derived from biopsies of donor paraffin-embedded specimens obtained from various subjects. The purpose of fabricating tissue microarrays is predominantly economic, facilitating a reduction in costs while simultaneously enabling the examination of a substantial number of specimens through a single assay, thus also reducing the time required for microscopic analysis. These specialized paraffin-embedded tissue blocks are used exclusively for research purposes [21]. Given the necessity for larger lung tissue specimens in this investigation, we judiciously adapted the tissue microarray construction methodology to correspond with our specific requirements. In the following section, we will briefly outline the procedure used in the construction of the tissue microarrays.

The encircled histopathological slides were superimposed onto the donor paraffin-embedded lung tissue to ensure that the biopsies would be extracted from a representative lung region comprising both alveolar and vascular structures. The tissue extraction was performed utilizing a hollow needle. The biopsy needle was introduced at an oblique angle into the specified region of the paraffin-embedded lung tissue, securing a cylindrical specimen measuring approximately 5 mm. To create a tissue microarray, all these lung fragments were re-embedded in two paraffin-embedded blocks, one designated for each medical center. As previously mentioned, the construction methodology of the tissue microarray has been modified to suit our specific requirements. Instead of incorporating the fragments in a vertical orientation, we have chosen a longitudinal arrangement to facilitate a more thorough examination of the lung surface. The precise positioning and codification of each segment of pulmonary tissue were meticulously executed to facilitate accurate identification during subsequent assessments.

#### 2.2.3. Immunohistochemical Analysis

For the evaluation of pulmonary microvascularization and the media layer of the vascular walls, an immunohistochemical analysis was conducted using an automated method to detect the presence of CD34 and SMA. These evaluations were performed on the tissue microarrays that had been previously created. The sections were made with a thickness of 4 μm. Once the sections were cut, they were mounted to the slide and placed in an oven for one hour. Subsequently, the sections were placed in PT-link (Dako), with antigen retrieval at a high pH and an incubation period of 30 min at 97 degrees Celsius. When the water temperature reached 65 degrees Celsius, the slides were removed and then immersed in a wash buffer for 10 min, allowing them to equilibrate to ambient temperature. Subsequently, the slides were treated with a blocking peroxidase solution for a period of five minutes, followed by thorough washing in three sequential baths of wash buffer. The specific antibody was then applied, which required an incubation period of twenty minutes. Thereafter, the slides undergo three successive washes in wash buffer, followed by HPR for twenty minutes. Following another twenty-minute interval, the slides underwent three additional washes in the wash buffer. The substrate was prepared in conjunction with DAB and then incubated for five minutes, after which three additional successive washes in the wash buffer were performed. The slides undergo a counterstaining process with hematoxylin for one minute, followed by a dehydration process achieved through a systematic immersion in a series of baths, ranging from 96% alcohol to absolute alcohol. Ultimately, the slides were clarified through three washes in xylene. The slides were loaded into the automated glass coverslipper, and thereafter, they were set for analysis under the optical microscope. CD34 acts as an immunohistochemical marker that delineates endothelial cells, whereas SMA serves as a cytoplasmic or membranous marker that accentuates smooth muscle fibers. Table 2 outlines the details related to the immunohistochemical markers, the clones selected, and additional parameters recommended by the manufacturers.

#### 2.2.4. Digital Image Analysis and Microscopic Evaluation

Microscopic analysis of the immunohistochemical markers was performed using the Nikon Eclipse Si trinocular microscope, which incorporates an optical system designed for infinite correction of chromatic aberrations. This microscope is equipped with a digital camera specifically designed for microscopy, the M-Shot digital camera MSX2, which offers 12.5 megapixels and a resolution of 4088 × 3072 pixels. From each case, representative micrographs were captured at a magnification of 200×, and the image processing was carried out using the Mshot Digital Imaging Software Version 1.1.6.

The assessment of CD34 expression was performed by taking 5 measurements on the captured microscopic images. The quantification of capillaries within the alveolar septum was carried out by counting the capillaries from 5 alveoli with diameters ranging between 60.0 μm and 120.0 μm (+/−5 μm). We designated as capillary structures all tubular formations exhibiting circumferential or semicircumferential immunostaining with CD34. After recording the data, the minimum and maximum number of capillaries, along with the average of the five determinations, were documented in the Excel database.

**Figure 1:**
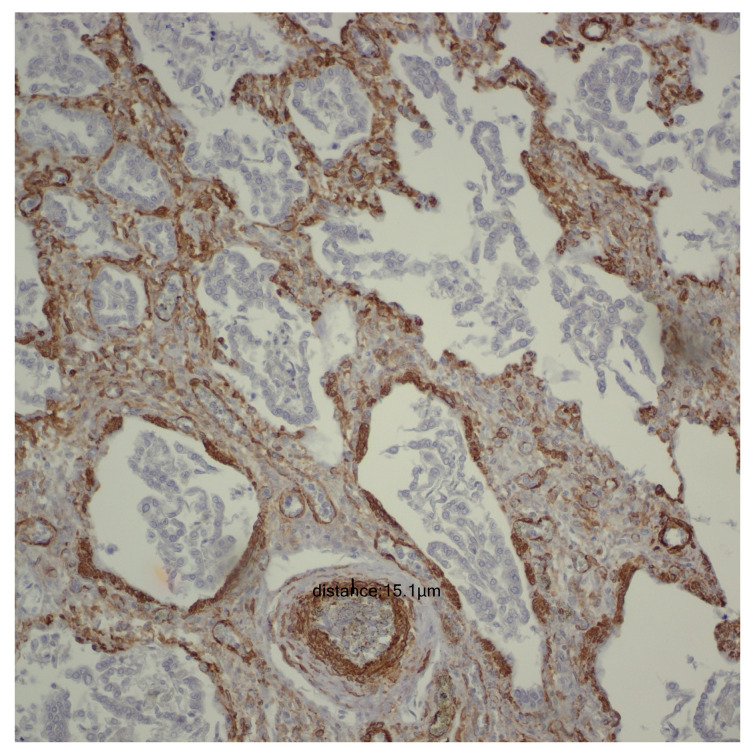
Smooth Muscle Actin (SMA) immunoreactivity at 200× magnification, illustrating the thickness of the vascular media.

For the evaluation of the media layer of the vascular walls, a vertical line was drawn between the highest point and the lowest point, recording the values obtained for each individual case. To prevent inaccuracies in measuring the arteriolar media, tangentially captured vascular structures were deliberately avoided, with measurements taken exclusively at the level of transversely captured vascular structures (Figure 1).

### 2.3. Statistical Analysis and Data Interpretation

All data gathered in the database was analyzed both descriptively and analytically, utilizing IBM SPSS software, version 28, and Microsoft Office Excel 2021 Pro Plus. Quantitative parameters with a uniform distribution were described by the mean and standard deviation and subsequently compared using the ANOVA test.

For each set of parameters, a null hypothesis was formulated, suggesting that there exists no significant disparity or correlation between the two parameters, while an alternative hypothesis posited the presence of at least one statistically significant difference or association between them. The null hypothesis was rejected based on the *p*-value. If the *p*-value was less than 0.05, the null hypothesis was rejected, and the alternative hypothesis was accepted, indicating that there are statistically significant variances or correlations between the analyzed parameters. The Pearson correlation coefficient was used to quantify the strength and direction of the linear relationship between two variables by explaining the extent and orientation to which one variable aligns with the other variable of interest.

Thus, the correlation coefficient was interpreted as indicated in Table 3 [22].

## 3. Results

**Figure 2:**
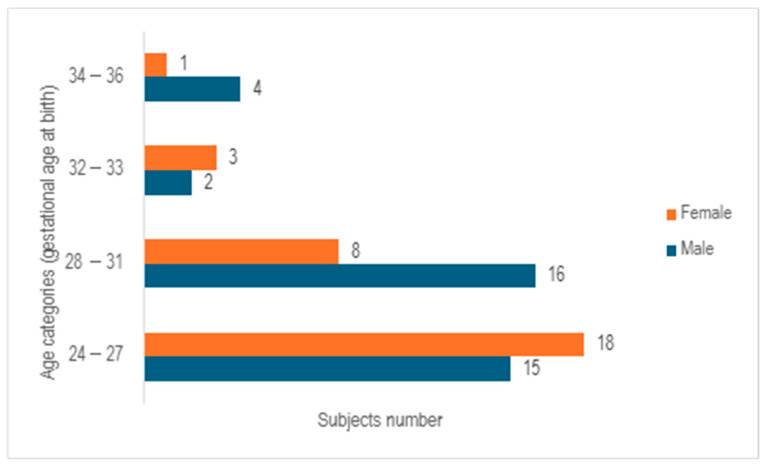
Distribution of subjects by age category and gender.

After analyzing the 67 cases examined in the study, it was observed that 55.2% of the cases involved male neonates, whereas 44.8% pertained to female neonates, with a median gestational age at birth of 28 weeks (ranging from 23 to 35 weeks). Following the categorization of subjects into 4 age cohorts based on gender, a predominance of females was noted in the age bracket of 24–27 weeks of gestation, while a prevalence of males was observed in the 28–31 weeks of gestation group. In contrast, a relatively even distribution between the genders was identified in the 32–33 weeks of gestation category. The group of infants born at 32–33 weeks of gestation and 34–36 weeks of gestation exhibited the lowest numbers, comprising only five subjects in each cohort (Figure 2).

After evaluating the birth weight and weight at demise, it was noted that the mean birth weight was 1116.34 g (ranging from 250 to 3850 g), while at demise, it was recorded at 1409.91 g. It is important to highlight that the exceptional birth weight of 3850 g was attributed to the presence of hydrops fetalis, resulting in a misleadingly elevated weight for a preterm infant. Among the entire cohort examined, 20.9% (n = 14) displayed intrauterine growth restriction. Out of these 14 cases, 11 occurrences (78.57%) were identified in mothers suffering from gestational hypertension, with 4 of them (28.57%) advancing to develop preeclampsia.

If we analyze the modality of delivery, a substantial majority of the participants (62.7%) were delivered via cesarean section, with cephalic presentation being the predominant fetal position at birth (68.65%) and the transverse position the least prevalent (8.95%).

**Figure 3:**
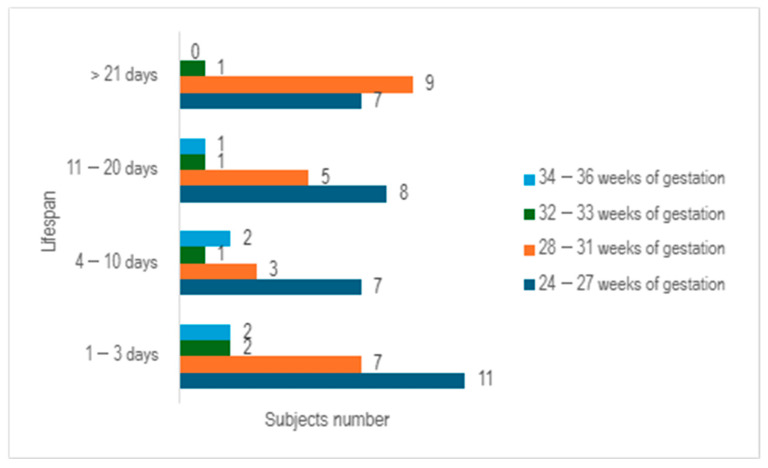
Distribution of subjects by age category and lifespan.

Upon examining the survival rates among different age groups, it becomes evident that the largest proportion of premature infants survived for a period ranging from 1 to 3 days (n = 22), with the majority falling within the 24–27 weeks of gestation category. The premature infants who managed to survive for durations of 4–10 days, as well as those lasting from 11 to 20 days, exhibited a relatively similar number of cases (n = 13 vs. n = 15) (Figure 3).

If we examine the gestational age cohorts at birth and scrutinize their maternal characteristics in relation to gestational hypertension and maternal infections, we would observe that within the 24–27 weeks gestation category, 15.15% of mothers (n = 5) exhibited pregnancy-induced hypertension, with 40% of these cases (n = 2) progressing to preeclampsia. In contrast, within the 28–31 weeks gestation cohort, 33.33% of mothers (n = 8) experienced gestational hypertension, and 37.5% of these instances (n = 3) progressed to preeclampsia.

When we evaluate the prevalence of maternal infections across these two gestational age groups, it becomes evident that maternal infections were significantly more prevalent in the cohort of 24–27 weeks of gestation at birth, which corresponds to the second trimester of pregnancy. In this group, 42.42% (n = 14) experienced infections during pregnancy, with a concerning 87.71% (n = 12) of these cases accompanied by premature rupture of membranes, characterized by a mean duration of 140 h prior to delivery. In contrast, within the age group of 28–31 weeks of gestation at birth, corresponding to the third trimester of pregnancy, only 29.16% (n = 7) encountered maternal infections during pregnancy. Furthermore, 71.42% (n = 5) of this group presented with premature rupture of membranes, with a mean duration of 44.6 h preceding delivery.

**Figure 4:**
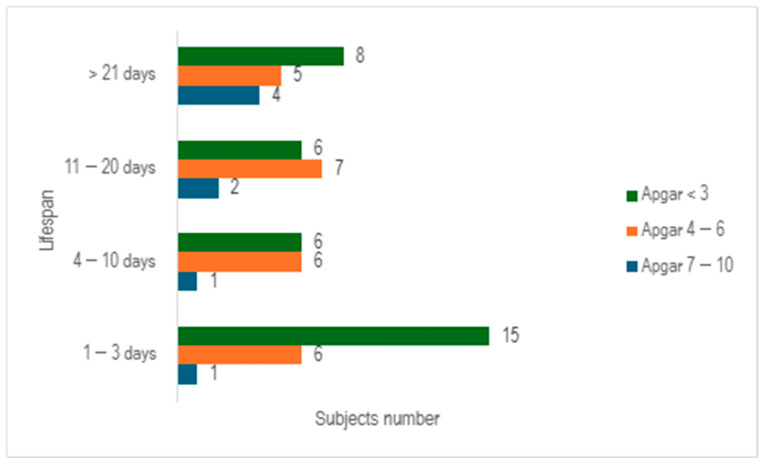
Distribution of subjects by lifespan and Apgar score at 1 min.

When we evaluated the study cohort in relation to lifespan and the Apgar score, stratifying the subjects into three distinct categories (scores below 3, scores ranging from 4 to 6, and scores exceeding 7), we discerned that a significant proportion of preterm infants exhibited an Apgar score below 3 at 1 min (n = 35), with nearly half of them succumbing within 3 days (n = 15) (Figure 4).

**Figure 5:**
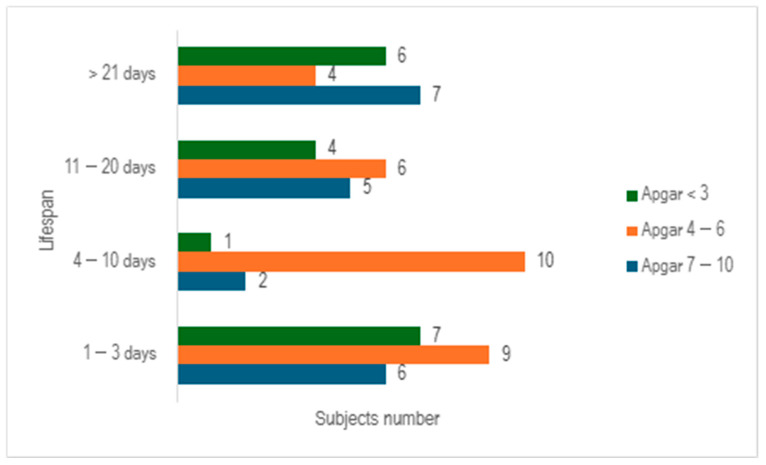
Distribution of subjects by lifespan and Apgar score at 5 min.

The assessment of the Apgar score at 5 min revealed an upward trend, with the majority of neonates scoring between 4 and 6 (n =29). Additionally, it was observed that 25.37% of the total cohort survived beyond 21 days (Figure 5).

Since medical data regarding the Apgar score at 10 min was not accessible to all participants in the study, an assessment of this determination could not be carried out.

If we consider oxygen therapy, all premature infants (100%) benefited from oxygen supplementation. The length of oxygen therapy (varying from 1 to 131 days) exhibited a direct relationship with the lifespan of these neonates (spanning from 1 to 149 days).

Additional interventions administered to these preterm infants can be found in Table 4.

The notable maternal characteristics of these infants are delineated in Table 5.

When we evaluated the number of pregnancies, one of the mothers experienced a total of 20 pregnancies, of which 19 ended in miscarriages. Of the entire cohort examined, 15 mothers experienced pregnancy-induced hypertension, with fewer than half of them progressing to preeclampsia (40%).

A significant proportion of cases did not derive benefits from antenatal corticosteroids (43.28%). This circumstance can be attributed to inadequate compliance among patients, with 18 individuals failing to undergo prenatal examinations. In contrast, certain cases involved critical medical or surgical situations, necessitating urgent cesarean delivery, or the patients presenting at the hospital in an advanced stage of labor.

**Figure 6:**
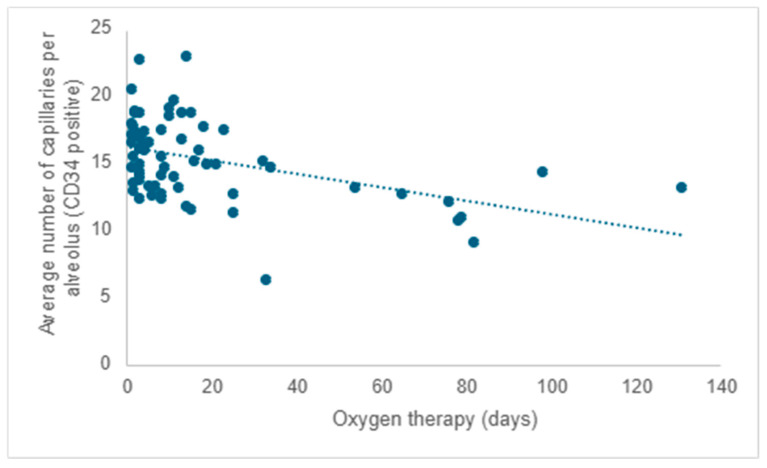
The influence of oxygen therapy on the mean number of capillaries per alveolus.

Starting from existing research, much of which was conducted on laboratory animals, and considering that all participants in the study received oxygen therapy, the researchers aimed to investigate the impact of oxygen therapy on pulmonary microvascular remodeling. Following the evaluation of the quantity of capillaries positive for CD34 (the mean value from five determinations), a negative correlation was observed between the average capillary count per alveolus and the duration of oxygen therapy. As the duration of oxygen therapy increased, there was a corresponding decrease in the number of capillaries, reaffirming the detrimental impact of this necessary intervention (r = −0.31; *p* < 0.001) (Figure 6).

When evaluating the mean number of capillaries (CD34 positive) across different age cohorts, it was noted that premature neonates who survived between 1 and 3 days exhibited an average of 3.43 more capillaries in comparison to those who survived beyond 21 days (95% CI: 1.061–5.811; *p* < 0.001). Furthermore, those who survived between 11 and 20 days displayed an average of 3.16 more capillaries compared to those who survived over 21 days (95% CI: 0.533–5.795; *p* = 0.01). Premature neonates who survived between 4 and 10 days exhibited no statistically significant differences in the number of capillaries per alveolus compared to other age groups.

In our examination of the mean capillary alveolar count in relation to gestational age cohorts at birth—specifically, 24–27 weeks of gestation, which corresponds to the second trimester, and 28–31 weeks of gestation, indicative of the third trimester—we did not identify any statistically significant correlation between these gestational age groups and their mean number of septal capillaries (*p* = 0.41).

Although pulmonary fibrosis is now less common among premature newborns than before the introduction of surfactant therapy and corticosteroid treatment, 10.44% (n = 7) of the neonates in the investigation exhibited this condition. Within this subset of premature neonates, there was an average decrease of 5.43 capillaries (CD34 positive) compared to newborns, with primary morphological changes characterized by alveolar simplification (95% CI: 3.40–7.44; *p* < 0.001).

**Figure 7:**
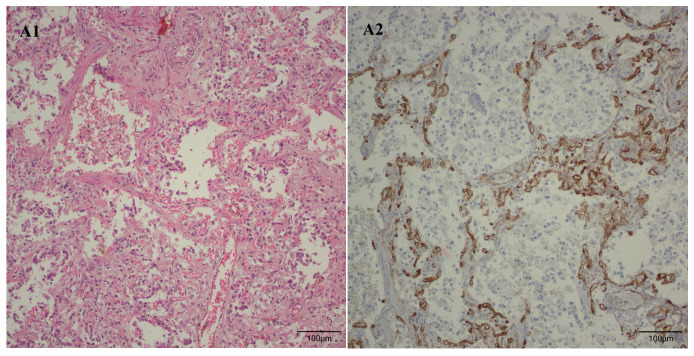

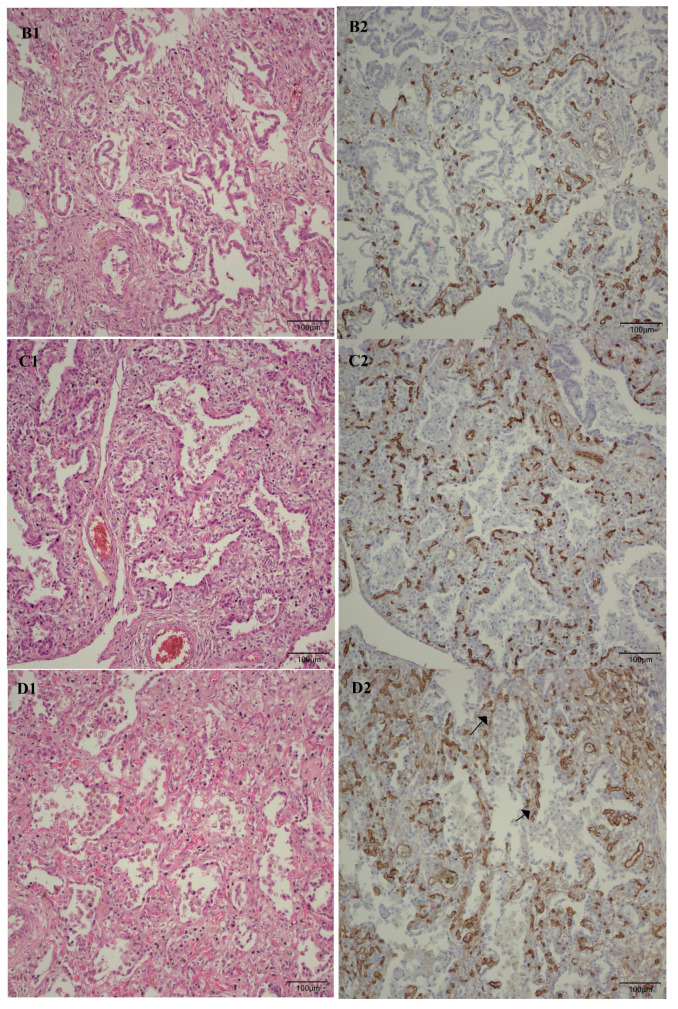

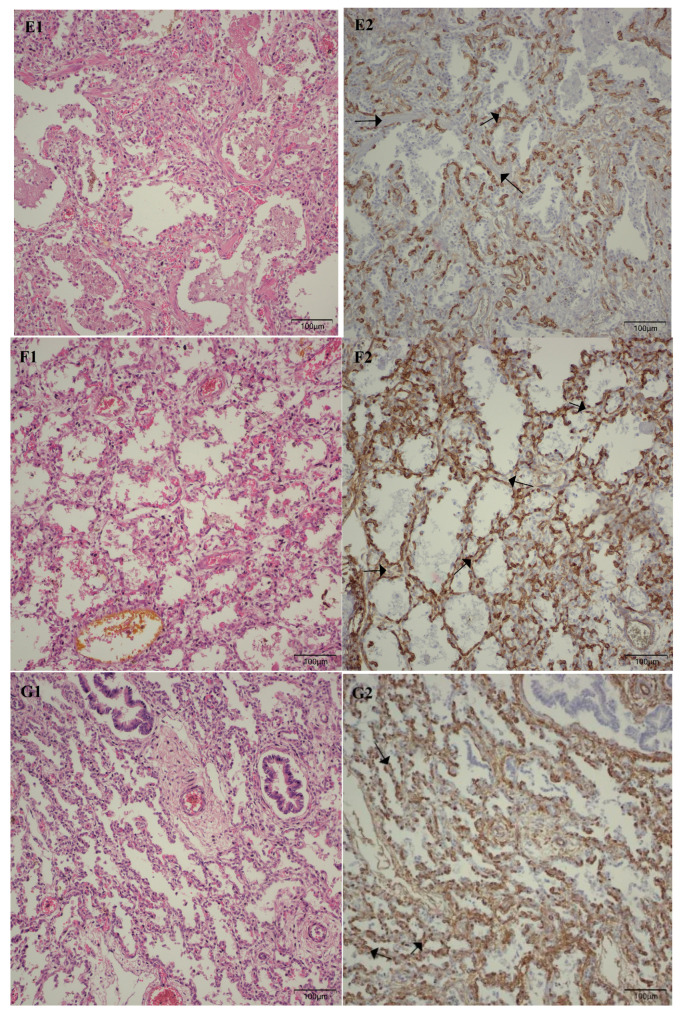
(A1* → G1*) Microscopic images in standard hematoxylin–eosin stain at a magnification of 200×. (A2* → G2*) Immunohistochemical staining for CD34, at a magnification of 200×. All images from A to E are derived from pulmonary samples of preterm neonates afflicted with pulmonary fibrosis, while images from F to G originate from preterm neonates exhibiting predominant morphological alterations characterized by alveolar simplification. (**A1**,**A2**) Lung tissue was obtained from a 29-week gestation neonate who survived for 149 days and underwent 82 days of oxygen therapy. (**B1**,**B2**) Lung tissue was obtained from a 26-week gestation neonate who survived for 45 days and underwent 33 days of oxygen therapy. (**C1**,**C2**) Lung tissue was obtained from a 26-week gestation neonate who survived for 93 days and underwent 78 days of oxygen therapy. (**D1**,**D2**) Lung tissue was obtained from a 25-week gestation neonate who survived for 111 days and underwent 79 days of oxygen therapy. (**E1**,**E2**) Lung tissue was obtained from a 29-week gestation neonate who survived for 131 days and underwent 131 days of oxygen therapy. (**F1**,**F2**) Lung tissue was obtained from a 28-week gestation neonate who survived for 16 days and underwent 13 days of oxygen therapy. (**G1**,**G2**) Lung tissue was obtained from a 27-week gestation neonate who survived for 39 days and underwent 34 days of oxygen therapy. The images A2 to E2 not only exhibit a reduced number of septal capillaries compared to the images F2 and G2, but also reveal an aberrant, disordered, and chaotic distribution of the capillaries. The arrows displayed in images D2 to G2 highlight the abnormal septal double layer of capillaries present in these premature infants. *All images from A1 to G1 and the corresponding immunohistochemical images from A2 to G2 are sourced from Dr. Raluca Chirculescu’s collection of cases.

Following the microscopic examination utilizing standard hematoxylin–eosin staining of the lung tissue from these premature neonates, we conducted a comparative analysis of the alveolar septum in premature infants afflicted with pulmonary fibrosis versus those exhibiting a predominant morphological alteration characterized by alveolar simplification. This analysis revealed a pronounced disparity in the thickness of these septa. In instances defined by pulmonary fibrosis, the alveolar septa demonstrated significant thickening and were abundant in dense connective tissue. Conversely, in cases characterized by alveolar simplification, the alveolar septa display a slender and delicate architecture, accompanied by a notable reduction in connective tissue. Upon analyzing the immunohistochemical images for CD34 that highlight the endothelial cells of vascular structures, two predominant characteristics become strikingly apparent. The first noteworthy observation pertains to the significant reduction in the number of septal capillaries observed in cases of pulmonary fibrosis, alongside the irregular distribution of these capillaries within the alveolar septum, starkly contrasting with the relatively uniform distribution of septal capillaries observed in cases characterized by alveolar simplification. The second prominent feature is the discernible septal double network of capillaries, observable in both premature neonates afflicted by pulmonary fibrosis and those whose primary morphological alteration is defined by alveolar simplification. An essential aspect to underscore is that this septal double network of capillaries was consistently present throughout the entirety of the study cohort, regardless of the gestational age at birth or the lifespan of these premature neonates. All these detailed microscopic and immunohistochemical characteristics are illustrated in Figure 7.

As illustrated in Table 5, 22.38% of mothers of preterm neonates exhibited gestational hypertension, whereas 8.95% exhibited preeclampsia. In this context, the aim was to investigate the possible correlation between these maternal conditions and pulmonary microvascular remodeling in preterm neonates. When assessing neonates born to mothers with gestational hypertension, we did not observe any statistically significant association (*p* = 0.70). However, in the case of preterm neonates born to mothers with preeclampsia, a notable disparity emerged. These infants exhibited an average reduction of 2.82 capillaries compared to those born to mothers unaffected by this pregnancy complication (95% CI: 0.32–5.32; *p* = 0.027).

In this investigation, we also examined the therapy of premature neonates with caffeine and pulmonary vasodilators. Although we did not establish a statistically significant correlation between caffeine administration and the number of alveolar capillaries (*p* = 0.09) or between the pulmonary vasodilators and the quantity of alveolar capillaries (*p* = 0.09), it is worth noting that the *p*-value approaches the threshold of 0.05, suggesting potential statistical significance. It is crucial to consider the possible limitations of the sample size in this study, which may lead to a reduction in statistical power.

Although the literature contains information regarding the correlation between maternal infections and pulmonary vascular remodeling, this study did not identify any association between maternal infection and pulmonary microvascularization, at least not in terms of the average number of septal capillaries (*p* = 0.28).

**Figure 8:**
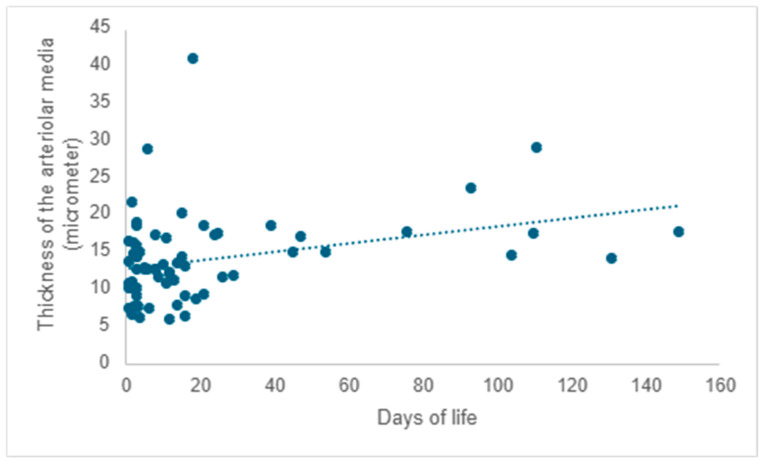
The impact of lifespan on the thickness of the arteriolar media.

After evaluating pulmonary microvascularization, we proceeded to examine larger vascular structures of the arteriolar type and assess the thickness of the arteriolar media by analyzing the SMA immunohistochemical marker (please refer to the evaluation protocol). We aimed to investigate whether the thickness of the arteriolar media was affected by the number of days of life and oxygen treatment, and we noted a positive correlation between them. As life expectancy rises, there is a tendency for an increase in the thickness of the arteriolar media (r = 0.22; *p* = 0.008) (Figure 8).

**Figure 9:**
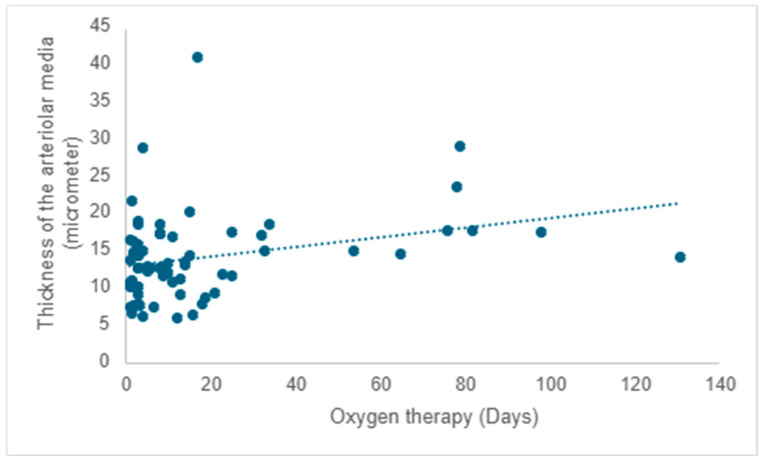
The impact of oxygen therapy on the thickness of the arteriolar media.

A positive correlation is evident between the duration of oxygen therapy and the thickening of the arteriolar media, signifying a rise in thickness with prolonged oxygen therapy (r = 0.20; *p* =0.017) (Figure 9).

It is widely recognized that some children diagnosed with bronchopulmonary dysplasia may develop pulmonary hypertension, a condition that histologically corresponds with arteriolar media hyperplasia. Hence, our study aimed to investigate the validity of this association within our sample cohort. The findings revealed a significant disparity of −5.31 in the thickness of arteriolar wall media between the group of children lacking histological markers of pulmonary fibrosis, in contrast to those manifesting distinctive histopathological signs of pulmonary fibrosis (95% CI: −9.84–−0.79; *p* =0.022).

As previously demonstrated, there was no apparent association between maternal infection during pregnancy and pulmonary microvascularization. In our ongoing exploration, we sought to investigate the potential correlation between maternal infections during pregnancy and arteriolar media thickness, as indicated by the immunohistochemical marker SMA. However, once again, no statistically significant correlation was found (*p* = 0.57).

Antenatal corticotherapy plays an important role in fetal lung maturation and in preparing the preterm newborn for the transition to extrauterine life. In this study, we also evaluated the vascular immunohistochemical parameters (CD34 and SMA) in premature neonates born to mothers who received antenatal steroids. By comparing these findings with those of preterm infants born to mothers who did not receive corticosteroids, no statistically significant correlation was observed (*p* = 0.9).

## 4. Discussion

As previously mentioned, the primary objective of this study was to evaluate the influence of antenatal risk factors, such as gestational hypertension, steroid treatments, and maternal infections, as well as postnatal risk factors, like incomplete lung development, oxygen therapy, and other treatments, on pulmonary vascular remodeling in premature neonates.

Pulmonary abnormal microvascularization was a constant microscopic feature observed throughout the entire cohort under investigation, regardless of the days of life or gestational age at birth. It primarily manifested as the retention of the double-layered capillary septa. An unusual vascular pattern in a full-term human newborn yet a characteristic vascular arrangement during fetal life, thus indicating morphologically that pulmonary vascular development has ceased to progress to a singular capillary vascular network that connects two alveolar spaces, despite the survival of some of these premature infants for nearly five months. Although the study cohort did not encompass a significant number of premature infants who developed pulmonary fibrosis, those who did experience this complication exhibited not only a markedly reduced mean number of septal capillaries in comparison to their counterparts but also an erratically disorganized distribution of these capillaries within the alveolar septa.

With the increase in lifespan, the length of oxygen therapy was extended, while the number of septal capillaries experienced a significant decline, particularly among neonates who had lived for more than 21 days and among those who had acquired pulmonary fibrosis. Over time, comparable observations regarding a reduction in the number of septal capillaries, as well as their chaotic distribution throughout the alveolar septum, have been reported by other researchers [1,23]. Oxygen therapy, although indispensable for the survival of these premature neonates, can have adverse effects on lung morphology and postnatal pulmonary development. Despite significant advancements in modern oxygen therapy techniques, a subset of these premature infants still experiences notable repercussions from this type of treatment. Why some premature neonates experience favorable outcomes while others contribute to the neonatal mortality rate remains an unresolved question.

Another important risk factor in the development of chronic lung disease is gestational hypertension and preeclampsia. In a study conducted by Matyas et al., they examined the effects of preeclampsia on the outcomes of preterm infants, and it was found that the severity of respiratory distress syndrome was twice as prevalent in the cohort of premature infants born to mothers who developed preeclampsia [24]. There are studies in the literature that discuss a potential protective effect of preeclampsia against respiratory distress syndrome, attributed to the acceleration of lung maturation. This effect may be mediated by an elevation in fetal serum cortisol levels. However, this protective mechanism was not observed in the study conducted by Wen et al. Instead, their findings, in line with the research by Matyas et al., indicated an elevated susceptibility to severe forms of respiratory distress syndrome [24,25]. Evaluation from our research showed that neonates born to mothers affected by preeclampsia exhibited a diminished quantity of septal capillaries compared to neonates born to unaffected mothers (*p* = 0.027). It is difficult to ascertain whether this correlation is genuine or instead attributable to the unique characteristics of this specific subset of neonates, given that these individuals had a lifespan ranging from 3 to 149 days and underwent oxygen therapy for periods extending from 3 to 82 days. Since we have already established that the duration of oxygen therapy has an inversely proportional effect on the number of capillaries at the alveolar septum, we must ask ourselves whether the relationship of preeclampsia in this context may simply serve as a confounding factor, thereby necessitating further investigations to ascertain the veracity of this association.

Corticosteroid therapy has been utilized and is still used to improve pulmonary function and decrease the requirement for postnatal ventilation [26]. The administration of antenatal steroids has been shown to decrease the thickness of the alveolar septum, which constitutes the alveolocapillary membrane, and accelerate the production of surfactant [27]. Considering the premise that antenatal corticosteroids may trigger morphological modifications in the alveolar septum, we set out to ascertain whether this association also involves changes in the pulmonary vascular network. Our study did not reveal any alterations in the septal capillary density or in the arteriolar media thickness, indicating that, irrespective of the morphological changes observed at the alveolar septa, they do not relate to the pulmonary microvasculature or the thickness of the vascular walls.

Although in this study, we were unable to establish a correlation between the presence of antenatal maternal infection and the presence of pulmonary vascular changes, there are studies in the literature describing the thickening of vascular walls, primarily due to changes affecting the arteriolar media and adventitia [28]. Willems et al. conducted a study involving preterm lambs that had been exposed to Ureaplasma parvum in utero 24 days prior to premature delivery, in addition to the administration of lipopolysaccharide 7 and 2 days before birth. The aim was to investigate the potential impact of intrauterine exposure to this pathogen or lipopolysaccharide during the canalicular phase of lung development on pulmonary vascular changes and lung inflammatory responses. The study findings revealed minimal changes in the vascular wall-to-lumen ratio, with a *p*-value approaching statistical significance (*p* = 0.06) and the presence of adventitial fibrosis [29]. Although this result did not reach statistical significance, it approached the threshold closely, unlike the result of our investigation, where we cannot assert any potential statistical significance (*p* = 0.57). This discrepancy in the results may be influenced by at least two fundamental factors. The primary potential reason pertains to the assessment protocol in which we meticulously examined the thickness of the arteriolar media while disregarding the vascular lumen, unlike Willems et al., who calculated a ratio between the vascular lumen and the thickness of the vascular wall. The second possible explanation concerns the classification of maternal infections. As previously stated, in her study, Willems established an animal model of chorioamnionitis by inducing chronic amniotic exposure in pregnant sheep to Ureaplasma parvum and/or lipopolysaccharide. In our research, we considered maternal antenatal infection, all cases documented in medical records with a history of infections, and the occurrence of premature rupture of membranes, regardless of bacteriological, morphological, or clinical evidence of chorioamnionitis. Looking at the data in this context, it is evident that the circumstances surrounding the evaluation of the vascular walls differ. This suggests the potential scenario in which some of the mothers of the deceased infants examined in our research may not have displayed intra-amniotic infection.

The vascular wall is composed of three distinct layers. Proceeding from the innermost layer of the vessel to the outermost, we refer to them as the intima, media, and adventitia. In cases of pulmonary hypertension, all three layers are affected, with the most prominent alterations manifesting in the media [30,31]. Prematurity is associated with an increased likelihood of developing pulmonary hypertension [32]. While it can arise in the absence of a chronic lung disease, such as bronchopulmonary dysplasia, pulmonary hypertension is more frequently observed in this population, especially among its more severe forms [33,34]. In experimental studies utilizing animal models, especially rodents, exposure to hyperoxia has been identified as a factor that induces an increase in the thickness of the walls of the pulmonary arterioles. Like previous research, our study demonstrated that the increase in the thickness of the arteriolar media manifested a direct association with lifespan and, by extension, with the sustained application of oxygen therapy. A similar trend in arteriolar media thickness was observed in pediatric patients who developed pulmonary fibrosis, suggesting that a subset of these infants exhibited heightened vascular tone and elevated vascular resistance, both of which are linked to pulmonary hypertension.

Even though these data originate from premature neonates who have succumbed to a severe pulmonary condition resulting from inadequate lung maturation, some persisting for nearly five months, it is evident that the majority of established and verified findings from experimental investigations in laboratory animals align with those observed in our study cohort. However, nowadays, a significant portion of premature newborns survives into adulthood. It is challenging to determine whether similar structural changes occur in the lungs of these individuals. It is plausible that certain modifications may manifest on a more subtle scale, along with the possibility that these structural modifications remain unrecognized and unexamined.

### Study Limitations

The primary limitation of the study lies in the small area of lung tissue examined, which was approximately 5 mm for each case. Despite this limitation, we meticulously stratified the measurable parameters to minimize potential errors in interpretation, and we can assert that at least some of our findings align with those acquired by other researchers over time.

Another notable constraint was that the data were acquired from deceased subjects, suggesting that the findings may be specific to individuals with advanced lung disease. Therefore, these findings may not be generalizable to the surviving pediatric population lacking clinical signs of lung impairment.

A third limitation of the study was the omission of an assessment of arteriolar media thickness in relation to gestational age at birth. As a result, the study was unable to identify whether certain gestational age cohorts demonstrated an elevated susceptibility to the hypertrophy of the arteriolar pulmonary media.

The fourth limitation pertains to the study’s sample size, which may be insufficient for achieving statistical significance, especially regarding specific parameters under investigation, such as the potential correlation between the number of alveolar capillaries and the administration of caffeine and pulmonary vasodilators, where the *p*-value approaches the upper threshold of 0.05.

## 5. Conclusions

Premature birth remains a significant concern in today’s society. While modern neonatal intensive care units fight to save the lives of premature infants at incredibly small gestational ages, these efforts are accompanied by considerable implications. Although prenatal factors such as gestational hypertension, antenatal steroids, and maternal infections may impact intrauterine fetal lung maturation, our investigation reveals that none of these factors significantly affect the subsequent development of postnatal pulmonary vascularization. While the presence of preeclampsia was correlated in this study with a decrease in the mean number of septal capillaries, further investigations are necessary to determine whether this maternal condition genuinely affects postnatal pulmonary vascular remodeling.

Regarding postnatal factors, two important factors significantly impact postnatal pulmonary vascular remodeling: incomplete pulmonary development and oxygen therapy. This research revealed the implications of the premature cessation of lung development on pulmonary microvascularization. Such disruption impairs the transformation of the septal capillaries’ dual-layer configuration into a single-layer capillary network, which is essential for the efficient optimization of gas exchange across the alveolocapillary membrane. Furthermore, oxygen therapy, commonly utilized in neonatal intensive care units, exerts a time-dependent adverse impact on the density of capillary networks within the alveolocapillary membrane.

An additional structural alteration observed in these premature neonates was the increased thickness of the arteriolar media, which correlated with lifespan and the extent of oxygen therapy. The same morphological observation was also noted in infants who developed pulmonary fibrosis, suggesting a predisposition to pulmonary hypertension in these infants.

In light of these findings, neonatologists and pediatricians must remain alert that incomplete pulmonary development, in conjunction with prolonged oxygen therapy, may impede the maturation of postnatal pulmonary microvascularization. Further studies are needed to determine whether this phenomenon is transitory or permanent.

## Figures and Tables

**Table 1:** Risk factors that can influence the development of bronchopulmonary dysplasia.

Risk Factors	
Antenatal	Genetic factors Absence of antenatal steroid therapy Intrauterine exposure to smoking GH and PE Maternal infections Hypoxia
Postnatal	Incomplete lung development Oxygen therapy Infections

GH: gestational hypertension; PE: preeclampsia.

**Table 2:** Technical specifications of the antibodies utilized.

Antibody	Clone	Dilution	Pretreatment (PT)	External/Internal Control
CD34	Dako/QBEnd 10	No dilution (RtU)	Dako PT Link	Endothelial cells
SMA	Dako/1A4	No dilution (RtU)	Dako PT Link	Smooth muscle tissue

CD: Cluster of Differentiation; SMA: Smooth Muscle Actin; RtU: Ready-to-Use.

**Table 3:** The interpretation of the Pearson correlation coefficient.

Correlation Power	Direct Correlation	Indirect Correlation
Perfect	r = (+1)	r = (−1)
Very strong	r ranges from (+0.90) to (+0.80)	r ranges from (−0.90) to (−0.80)
Moderate	r ranges from (+0.70) to (+0.60)	r ranges from (−0.70) to (−0.60)
Fair	r ranges from (+0.50) to (+0.30)	r ranges from (−0.50) to (−0.30)
Poor	r ranges from (+0.20) to (+0.10)	r ranges from (−0.20) to (−0.10)
None	r = 0	r = 0

r: the Pearson correlation coefficient.

**Table 4:** Therapeutic interventions implemented.

Administered Therapies:	Subjects Number
Surfactant therapy	45/67 (67.16%)
Antibiotic therapy	66/67 (99.50%)
Caffeine therapy	38/67 (56.71%)
Pulmonary vasodilator therapy	19/67 (28.35%)
Blood transfusions	56/67 (83.58%)
Immunoglobulins	23/67 (34.32%)

Data are presented as absolute numbers and percentages.

**Table 5:** Demographic and clinical characteristics of mothers of preterm infants included in the study.

Parameter	Variable	
Maternal age	31.52 ± 6.870 * (ranging from 16 to 45 yr)	
Marital status	Married Unmarried/Divorced	n = 38 (56.71%) n = 29 (43.28%)
Residence	Urban Rural	n = 30 (44.77%) n = 37 (55.22%)
Fetal monitoring during pregnancy	Yes No	n = 18 (26.86%) n = 49 (73.13%)
The number of pregnancies	3.09 ± 6.870 * (ranging from 1 to 20)	
The number of births	1.93 ± 1.222 * (ranging from 1 to 6)	
Antenatal steroids	Yes No	n = 29 (43.28%) n = 38 (56.71%)
Cervical cerclage or pessary	Yes No	n = 7 (10.44%) n = 60 (89.55%)
PROM	Yes No	n = 18 (26.86%) n = 49 (73.13%)
PROM (time)	28.766 ± 105.948 * (ranging from 0 to 696) ^a^	
Maternal infections during pregnancy	Yes No	n = 25 (37.31%) n = 42 (62.68%)
Preexisting hypertension during pregnancy	Yes No	n = 1 (1.49%) n = 66 (98.50%)
Gestational hypertension	Yes No	n = 15 (22.38%) n = 52 (77.61%)
Preeclampsia	Yes No	n = 6 (8.95%) n = 61 (91.04%)
IVF	Yes No	n = 3 (4.47%) n = 64 (95.52%)
Gestational diabetes	Yes No	n = 1 (1.49%) n = 66 (98.50%)
Anemia during pregnancy	Yes No	n = 6 (8.95%) n = 61 (91.04%)
Stillbirth	Yes No	n = 2 (2.98%) n = 65 (97.01%)

*: mean ± standard deviation; n: subject number; PROM—premature rupture of membranes; ^a^: hours of premature rupture of membranes prior to delivery; IVF—in vitro fertilization.

## Data Availability

The raw data supporting the conclusions of this article will be made available by the authors on request due to privacy, legal or ethical reasons.
